# Supplementary material for: Analyzing the sensitivity of quantitative 3D MRI of longitudinal relaxation at very low field in Gd-doped phantoms
Source: PLoS One. 2023 May 5;18(5):e0285391. doi: 10.1371/journal.pone.0285391 (PMC10162526; doi:10.1371/journal.pone.0285391)
Supplement: S1 File — Additional details on data pre-processing, working assumptions and ANOVA results. (DOCX) [file pone.0285391.s001.docx]

**SUPPLEMENTARY INFORMATION**

**Methods**

**Data pre-processing**

Different pre-processing procedures were applied to the data collected by the four scanners. Firstly, data obtained from the VLF scanner was processed to improve the SNR while minimizing the impact on the contrast. VLF images were recorded through 4 averages only, to avoid extremely long recording duration and allow comparison with the other scanners. VLF magnitude images were obtained from the raw data, which were complex values in 3D Fourier space, through the application of a Tukey filter before the Fourier transform. Then, to further increase the SNR, a denoising procedure through wavelet decomposition (‘db5’, Matlab, The Mathworks) and a moving average on the nearest pixels was applied on the reconstructed images.

The other commercial scanners directly produced magnitude images. All these images were resized to fit the voxel size of the VLF images (voxel sizes are listed in Table 1). Moreover, to compare the images obtained as a function of the T_R_, we rescaled the images at 0.2 T and 3 T since the internal signal scaling was not constant over the different T_R_ in these scanners. This is achieved through normalization by the average noise floor (for the 0.2 T images) or the noise STD (for the 3 T images), estimated over all the voxels outside the tubes. This procedure was necessary as we had no access to the raw data, as the 0.2 T and 3 T scanners' proprietary file format was access restricted, resulting in varied normalization post-processing. To this aim and for the subsequent analyses, a mask was obtained at each field from the image at the largest T_R_, using the Matlab function for segmentation of images *watershed,* that can identify homogeneous regions and their edges in the image and and remove the latter ones. For all the contrast analyses (see below) only the voxels comprised in the mask were considered, excluding the edge voxels since they could have contributions from the background noise floor.

**Control measurements on the assumption** **T_2_^*^ << T_R_**

In the manuscript (in the subsection *Contrast Analysis on R_1_ maps*) we gave an upper estimation for the expected T_2_^*^ values for the VLF experiments. As stated, that estimation is strictly valid during the acquisition window, when the reading gradient is on. After this window the signal, i.e. the transverse magnetization, is supposed to decrease with a different time scale since T_2_^*^ is determined by the intrinsic T_2_ and by the measurement field inhomogeneities.

We controlled that at the end of the acquisition window in the spin-echo sequence, the signals were already degraded down to the noise floor level, meaning that no residual transverse magnetization was present at the beginning of a new repetition. The reading gradient is not compensated after the acquisition window, then it basically acts as a spoiling gradient for the reading direction and prevents coherence pathways effects when acquiring different lines of k-space.

In Fig 1 we show the averaged signal during the acquisition from the central lines of the k-space along the reading direction (in blue), corresponding to the smallest phase encoding gradients amplitude and the most intense signal. Conversely, the averaged signal (in red) during the acquisition from the peripheral lines of the k-space along the reading direction (corresponding to the most intense phase encoding gradient amplitudes and the smallest signal) is representative of the noise floor. At the end of the acquisition window, the amplitudes of the two traces are similar and, in particular, this holds for the shortest T_R_ (shown on the left), demonstrating that our assumptions on T_2_^*^ << T_R_ are always correct in our case. No post-processing is applied to the signal and only an anti-aliasing filter (a Finite Impulse Response one) with a cut-off frequency equal to the Nyquist frequency (1.25 kHz) is acting during the acquisition.


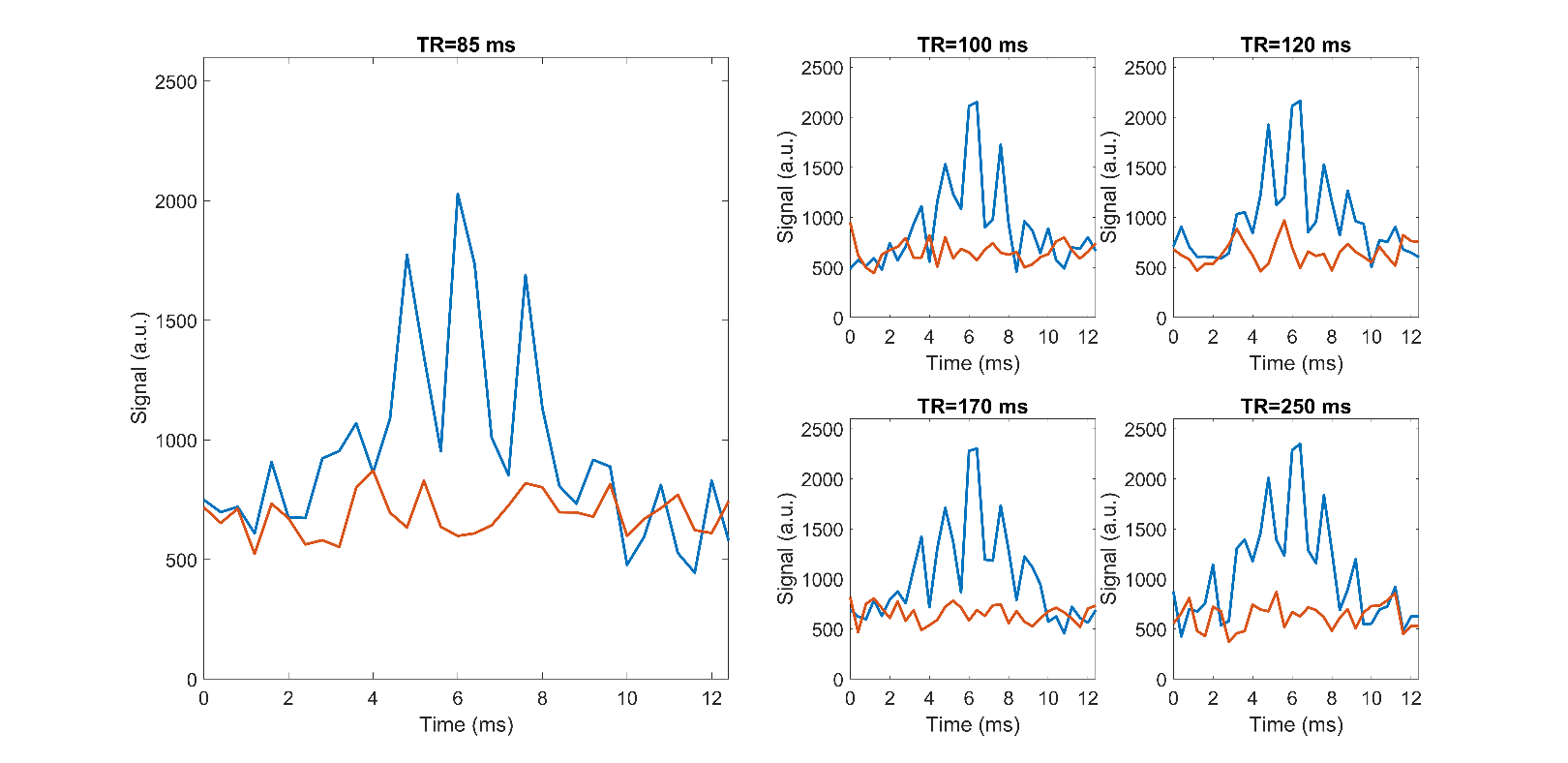


**Fig 1. Raw signals and noise floor.** Comparison between raw signals from k-space during the acquisition window at multiple T_R_. The shortest one, on the left, is the most interesting, demonstrating that our assumption on T_2_^*^ << T_R_ is always correct in our case. The blue line represents the mean signal from the 16 central lines of the 3D k-space, i.e. with almost null encoding shift in the two phase directions, while the red line represents the average of 16 peripheral lines of the 3D k-space in both the phase encoding directions, assumed as representative of the noise floor. Signals are reported as acquired, with only a finite impulse response anti-aliasing filter and no post-processing.

**Results**

**ANOVA on R_1_ values**

To quantitatively compare R_1_ values, we ran ANOVA which produced a significant main effect sample (F_4_ = 1313, *P* < 10^-100^), a significant categorical factor field ((F_3_ = 1575, *P* < 10^-100^) and a significant interaction (F_12_ = 44, *P* < 10^-50^). Bonferroni post-hoc on the categorical factor field confirmed that R_1_ at 8.9 mT is larger than the other fields (*P* < 10^-70^) and the same applies to 0.2 T with respect to the higher fields (*P* < 10^-50^). Notably, R_1_ at 1.5 T and 3 T were not significantly different. Moreover, Bonferroni post-hoc on interaction suggested that significantly different R_1_ values were associated to the different vessels for the VLF and the 1.5 T scanner (*P* < 2⋅10^-10^ and *P* < 0.03 respectively), while it was possible to disentangle only the three higher CA concentrations at 0.2 T and 3 T (*P* < 3⋅10^-7^). Moreover, for all the samples the R_1_ value significantly decreased with the measurement field up to 1.5 T (*P* < 3⋅10^-7^, except for *P* < 0.003 for the reference sample at 8.9 mT and 0.2 T), and did not differ from 1.5 to 3T. Bonferroni post-hoc on the categorical factor field confirmed that R_1_ at 8.9 mT is larger than for the other fields (*P* < 10^-70^) and the same applies to 0.2 T with respect to the higher fields (*P* < 10^-50^). Notably, R_1_ at 1.5 T and 3 T were not significantly different. Moreover, Bonferroni post-hoc on interaction suggested that significantly different R_1_ values were associated to the different tubes for the VLF and the 1.5 T measurements (*P* < 2⋅10^-10^ and *P*< 0.03 respectively), while it was possible to disentangle only the 3 higher CA concentrations at 0.2 T and 3 T (*P* < 3⋅10^-7^). Moreover, for all the samples the R_1_ value significantly decreased with the measurement field up to 1.5 T (*P* < 3⋅10^-7^, except for *P* < 0.003 for the reference sample at 8.9 mT and 0.2 T) and did not change between 1.5 and 3 T.

**Comparison between R_1_ maps and T_1_-w images**

*Fig 6 (main manuscript)*: ANOVA on R_1_ produced a significant categorical factor field (F_3_ = 1821, *P* < 10^-90^). Bonferroni post-hoc on the categorical factor field confirmed that R_1_ contrast at VLF is significantly larger than at other fields (*P* < 10^-14^), at 0.2 T is larger than at the two higher fields (*P* < 10^-14^), and at 1.5 T is larger than 3 T (*P* < 10^-8^).

Notably, while the R_1_ differences are clearly increasing as the field decreases, such an increase is not that clear in the image differences, where we obtained a significant categorical factor field (F_3_=180, *P* < 10^-90^), but it seems that although the contrast at VLF is higher than at high field (*P* < 0.05), it is lower than at 0.2 T (*P* < 10^-14^). The voxel value contrast at 1.5 T is again larger than at 3 T (*P* < 0.05).

*Fig 7 left (main manuscript)*: 1-way ANOVA with the field as categorical factor confirmed that R_1_ contrast changes with the applied field, significant categorical factor field (F_3_ = 996, *P* < 10^-100^), and Bonferroni post-hoc confirmed that R_1_ contrast at 8.9 mT is larger than at other fields (*P* < 3⋅10^-32^) and the same applies to 0.2 T with respect to the higher fields (*P* < 6⋅10^-17^), while no significant differences were found between 1.5 T and 3 T. R_1_ contrast was also significantly larger as the CA concentration increased, as assessed by the main effect sample (F_3_ = 545, *P* < 10^-100^) and Bonferroni post-hoc (*P*<10^-4^). We also obtained a significant interaction (F_9_ = 193, *P* < 10^-100^). Bonferroni post-hoc on interaction suggested that significantly increasing R_1_ contrast was associated to the increasing CA dilutions at VLF (*P* < 2⋅10^-15^), except for the highest CA concentration (*P* > 0.05). This effect was possibly induced by inadequate sampling in the low T_R_ range, as explained in the Discussion section. Notably, at the other fields, the contrast of Mh3000 and the reference sample and between Mh2000 and Mh3000 did not show a significant increase with the mean CA concentration as at VLF (we even note a significant decrease at 3 T – *P* < 0.01), suggesting a lower sensitivity to differences in low CA concentrations, consistently with results shown in Figure 4. The contrast at higher CA concentration is instead clearly detectable (*P* < 10^-24^).

*Fig 7 right (main manuscript)*: We note that the contrast between the 2 higher CA concentrations is negative, but this, as further discussed later, is due to a T_2_ effect, although significant interaction (F_12_ = 11, *P* < 10^-17^) from 1-way ANOVA with field as categorical factor over the normalized voxel values revealed that this contrast was significantly different from zero at VLF only (*P* < 3⋅10^-16^). The other contrasts are significantly different from zero at VLF (*P* < 0.015), while only the first contrast is significantly different from zero for 0.2 and 1.5 T (*P* < 3⋅10^-8^), while no difference is found at 3 T. 1-way ANOVA with field as categorical factor on voxel value contrast, similarly to the analysis ran on R_1_, produced a main effect of sample (F_3_ = 29, *P* < 10^-100^) and Bonferroni post hoc suggested that the contrasts from all sample are different (*P* < 10^-178^), although a clear trend was not found. Bonferroni post hoc on significant interaction (F_9_ = 198, *P* < 10^-100^) revealed that the contrast for the Mh3000-MhRef comparison was larger than the others (*P* < 10^-14^), except for 3 T. This contrast at VLF and 0.2 T was similar and was larger than at higher fields.
